# Supplementary material for: Diet Composition Affects Liver and Mammary Tissue Transcriptome in Primiparous Holstein Dairy Cows
Source: Animals (Basel). 2020 Jul 14;10(7):1191. doi: 10.3390/ani10071191 (PMC7401567; doi:10.3390/ani10071191)
Supplement: Supplementary file 1 [file animals-10-01191-s001.zip › animals-820137-supplementary/New folder/Figure S1.docx]

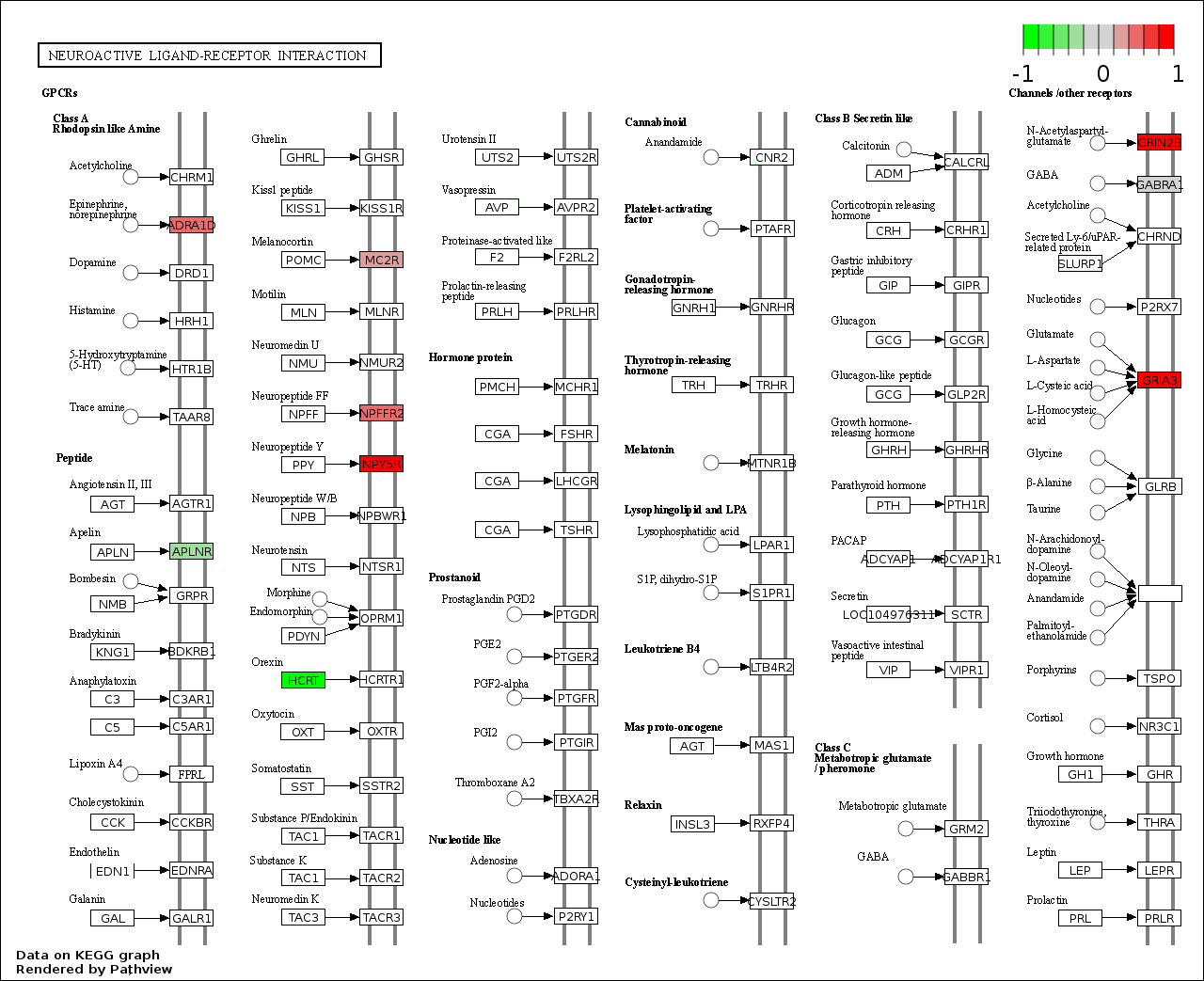


**Figure S1** Differently expressed genes (DEG) of mammary and liver of CS vs. MF in pathway of Neuroactive ligand-receptor interaction. Change direction of DEG are mapped by colors. Green colors represent that the genes were downregulated. Red colors represent that the genes were upregulated.
