# Supplementary material for: Diet Composition Affects Liver and Mammary Tissue Transcriptome in Primiparous Holstein Dairy Cows
Source: Animals (Basel). 2020 Jul 14;10(7):1191. doi: 10.3390/ani10071191 (PMC7401567; doi:10.3390/ani10071191)
Supplement: Supplementary file 1 [file animals-10-01191-s001.zip › animals-820137-supplementary/New folder/Table S1.docx]

**Table S1** Ingredients and chemical composition of experimental diets ǂ.

|  | Experimental diets | |
| --- | --- | --- |
|  | CS (F:C = 40:60)* | MF (F:C = 60:40) |
| Ingredients [g/100 g of DM] |  |  |
| Corn stover | 37.1 | - |
| Alfalfa hay | - | 28.4 |
| Corn silage | - | 26.5 |
| Chinese wile rye | - | 3.7 |
| Corn | 33.5 | 22.8 |
| Wheat bran | 3.0 | - |
| Soybean meal | 23.6 | 11.8 |
| Cottonseed fuzzy | - | 5.1 |
| Calcium phosphate | 0.4 | 0.6 |
| Limestone | 1.3 | - |
| NaCl | 0.5 | 0.5 |
| Mineral-vitamin mix† | 0.6 | 0.6 |
| Chemical composition [g/100 g of DM] |  |  |
| Dry matter content | 54.47 | 55.78 |
| Crude protein | 16.90 | 16.70 |
| Neutral detergent fibre | 41.01 | 44.18 |
| Acid detergent fibre | 21.15 | 26.06 |
| Ether extract | 1.58 | 2.24 |
| Calcium | 0.89 | 0.82 |
| Phosphorus | 0.21 | 0.31 |
| Net energy lactation (NEL)# [MJ/kg of DM] | 6.19 | 6.35 |

Notes: ǂ This table has been published in Han et al. (2014). For readers’ convenience, we supplemented it to this paper. *F:C, forage-to-concentrate ratio; †Containing (per kilogram dry matter of premix): vitamin A 2,000,000 IU, vitamin D 600,000 IU, vitamin E 10,800 mg, ferrum 4080 mg, copper 4989 mg, zinc 180 mg, manganese 17,500 mg, iodine 180 mg; cobalt 8805 mg; #Estimated based on chemical compositions and 24 h gas production of diets (Hohenheim gas test).
